# Surgical innovation, statistical analysis, and professional culture: thymectomy for myasthenia gravis, 1936–2016

**DOI:** 10.1017/mdh.2024.35

**Published:** 2025-01

**Authors:** Mark W. Weatherall

**Affiliations:** Stoke Mandeville Hospital, Aylesbury, UK

**Keywords:** Myasthenia gravis, Thymectomy, Thoracic surgery, Controversy, Clinical trials

## Abstract

This paper provides an account of a specific operation – the removal of the thymus gland (thymectomy) to treat the rare neurological condition myasthenia gravis – from its first performance in 1936, by the American surgeon Alfred Blalock, to the publication in 2016 of an international multicentre randomised controlled trial (RCT) of the technique. Thymectomy was the subject of a transatlantic controversy in the 1950s, in which the main players were the English surgeon Geoffrey Keynes, and American neurologists and surgeons from New York, Boston, and the Mayo Clinic. The resolution of this controversy involved the use of increasingly sophisticated statistical techniques, but also crucially other influences including the social transformation of thoracic surgery, and competition between the leading American centres. The consensus achieved after this controversy was challenged in the late 1970s, eventually prompting the implementation of a trial acceptable to twenty-first-century evidence-based medicine. This account will demonstrate that surgical innovation in the period covered required increasing attention to the statistical basis of patient selection and outcome evaluation; that the processes of technical innovation cannot be regarded as separate from developments in the professional culture of surgery, and that one of the consequences of these changes has been the gradual eclipse of the prestigious autonomous surgeon.

Of the many changes in medicine over the last two centuries, one of the most striking is the emergence of surgery to become, as Thomas Schlich puts it in his introduction to the *Palgrave Handbook of the History of Surgery*, ‘a universal, safe, and a certain extent even popular way of solving a whole variety of medical (and some non-medical) problems.’^1^ Over the last three decades, historians of medicine have become increasingly interested in the professional, social, economic, and technical factors that have contributed to this transformation.^2^ Much of this work has revolved around questions of surgical innovation: how was new surgical knowledge produced and disseminated? How did surgeons demonstrate the safety and utility of their operations? What did it mean to be a successful surgeon?

Word-of-mouth testimony was the traditional means of documenting and presenting new surgical techniques and building a surgical reputation. As technologies of the printed word developed and became more accessible in the seventeenth and eighteenth centuries, surgical case history became a means by which the reader could become a virtual witness to an operation and its outcome.^3^ In addition to this, as Warwick has shown, surgeons who were interested in new operations would often travel, singly or in groups, to leading centres to see new operations being done, and to learn the craft skills and tacit knowledge necessary to perform these operations successfully; ‘new surgical procedures’, Warwick comments, were ‘best propagated by direct demonstration’.^4^ Wilde and Hirst have shown how this process might work by examining the diaries of Archibald Watson, an early twentieth-century Australian anatomist and surgeon, who actively searched for improved techniques, and looked out for, and tried to learn from, his colleagues’ mistakes and errors, contributing to what Wilde and Hirst called ‘a creative process of invention’.^5^

The success, or otherwise, of operations was traditionally expressed in terms of surgical mortality and complication rates. This was, as Ulrich Tröhler comments, ‘important in its time for the self-regulation of surgery, as well as for winning the confidence of other doctors and patients alike’.^6^ Quantification of surgical outcomes was developed and increasingly utilised as a rhetorical device in the nineteenth century, most notably in debates surrounding Joseph Lister’s system of antisepsis.^7^ Other components of what would come to be known as randomised clinical trials (RCT), such as the use of controls (initially by the method of alternate allocation, then by randomisation), can be seen in some surgical studies in the 1920s and 1930s. At this time, however, the dominant mode of surgical knowledge production was the case series, a technique which served both to outline the potential of new operations, or new operative techniques, and also bolster the fame of a particular surgeon, and reinforce the myth of the surgeon as heroic innovator. Where disagreements arose (as Lerner has shown for William Halsted’s radical mastectomy operation, and Wilde for prostate surgery in 1930s Australia), debates were couched in terms of competing series, without necessarily achieving consensus.^8^

In the progressive, scientistic post-War era, however, lack of consensus was no longer acceptable. Definite answers were necessary, and the perceived successes of trials such as the 1948 Medical Research Council (MRC) randomized trial of streptomycin for tuberculosis were increasingly used to point out a difference in the standards of knowledge production between medicine and surgery. Surgeons were not slow to recognise the value of the techniques of the RCT, but there was also resistance, some nuanced, some not so, to its wholesale importation into surgery.^9^ On the one hand, the value of new surgical techniques or technological innovations was expected to be obvious and self-evident. On the other, it was claimed that surgical outcomes were inherently complex, depending on hundreds of tiny details of technique, as well as the input of multiple other medical professionals involved in pre-operative assessment, anaesthesia, intraoperative management, and post-operative care.^10^ Sometimes the institutional arrangements required to run co-operative multicentre studies with adequate statistical power were lacking: Timmerman and Valier have shown, for example, that attempts to develop ‘a co-ordinated scheme of investigation’ for cancer therapies in the 1960s failed, partly because of difficulty recruiting patients, and partly because it was found that comparable cases treated with usual procedures in different centres could not be matched in order to obtain a statistically significant evaluation of the results of treatment.^11^ In other cases, new techniques (such as laparoscopic [keyhole] surgery for gall bladder disease) continued to be introduced into widespread practice on the basis of case series, with RCTs only performed, almost as an afterthought, once the technique was already well established.^12^

Very few studies detail the transformation of the nature of surgical evidence over this period. This paper covers the history of one particular operation – the removal of the thymus gland (thymectomy) to treat the rare neurological condition myasthenia gravis – from its first performance in 1936 to the publication in 2016 of an international multicentre RCT of the technique, focussing on a transatlantic controversy in the 1950s.^13^ The resolution of this controversy involved increasingly sophisticated statistical techniques, but also other factors including the social transformation of thoracic surgery, and competition between leading American institutions. The thymectomy ‘black box’ was reopened in the late 1970s, eventually prompting (very late in the day) the implementation of an RCT on the topic. As a consequence of this process of revision and reassessment, we will see that, as Tröhler has suggested, the therapeutic evaluation of surgery required ever widening integration of information from other disciplines, leading to the subsummation of the prestigious autonomous surgeon within a broader collaborative team.

## Myasthenia, the thymus, and early American experiences of thymectomy 1936–50

Myasthenia gravis was a strange and elusive disease. It did not exist before the turn of the twentieth century. It does not appear, for example, in William Gowers’ definitive *Manual of Diseases of the Nervous System*, published in the 1880s and 1890s.^14^ In the subsequent two decades, however, several cases appeared in the neurological literature of patients experiencing fluctuating muscular weakness, on occasions sufficiently severe to cause death, without any obvious pathological changes to be seen in the muscles *postmortem.* In 1933, the tentative, provisional nature of the disease was caught by the neurologist Russell Brain in the first edition of his textbook, in which he described it as a ‘chronic disease with a tendency to remissions and relapses, characterized by abnormal muscular fatiguability, which for a long time can be confined to, or predominant in, an isolated group of muscles, and is later associated with a permanent weakness and sometimes wasting of some muscles’.^15^ The inconsistent nature of the symptoms often led them to be dismissed as functional or psychosomatic. It was perhaps not until after ‘the miracle at St Alfrege’s’ (the demonstration in 1935 by Dr Mary Broadfoot Walker [1888–1974] that neostigmine could temporarily reverse the effects of the disease) that myasthenia became firmly characterised as a disorder of the neuromuscular junction, albeit one with no known pathology and only limited treatment.^16^

Whilst the muscles of those who had died of myasthenia were normal at *postmortem*, a common finding was the presence of a persistent thymus gland. The thymus gland was a small collection of lymphoid tissue sitting in the chest behind the sternum; it was known to be present in children, but in most cases atrophied in later life. The connection, if any, between myasthenia and the thymus gland was unclear, but a small number of surgeons postulated that its removal (thymectomy) might be beneficial for patients with myasthenia, particularly as other options were limited.^17^ The first reported thymectomy was performed at the Vanderbilt Hospital in Nashville, Tennessee, by Alfred Blalock (1899–1964) in 1936. Blalock presented the case to a meeting of the American Surgical Association in Hot Springs, Vermont in May 1939, and an account was published in the journal *Annals of Surgery* in October of that year.^18^ Blalock moved to Johns Hopkins Hospital in Baltimore, publishing a series of six patients in 1941,^19^ increasing to twenty by 1944. Seventeen patients survived surgery; of these, three were well, five were ‘considerably improved’, five were ‘moderately improved’, three were ‘unchanged’, and one had died. ‘The early and sustained improvement which has been shown by some of these patients’, Blalock commented cautiously, ‘makes it difficult to escape the conclusion that thymectomy was at least partly instrumental in causing the alteration’. These patients were incorporated into the next publication from Johns Hopkins, by Abner McGhee Harvey (1911–98), comprising thirty-two patients and published in 1948; Harvey concluded that the data were insufficient to come to a judgement about the effectiveness of the intervention.^20^ Five years later David Grob (1919–2008) presented data on 202 Hopkins’ patients, of whom forty-four had undergone thymectomy, and forty irradiation of the thymus. The difference between the outcomes of the thymectomy patients and the controls was, Grob stated, ‘disappointingly small’.^21^

In 1938, even before Blalock’s case had been presented and published, the neurologists Henry Viets (1890–1969) and Robert Schwab (1904–72) were encouraging their surgical colleagues at the Massachusetts General Hospital to take up the operation; they started doing it more regularly beginning in December 1941, publishing a series of fifteen patients in 1945 (‘we are in the experimental stage’).^22^ Somewhere around this stage the team temporarily ceased recommending the operation. The English surgeon Geoffrey Langdon Keynes (1887–1982) stated at a meeting held in London in 1946 that Viets had done this because five out of his sixteen patients had died; Keynes speculated that this might have been due to the high incidence of tumours in Viets’ patients.^23^ Writing a decade later, Schwab and Viets recalled their discouragement, ‘but for some reason which neither of the authors… can remember, we kept our surgical colleagues interested and subsequently referred patients into the hospital for thymectomy’, and by 1947 they had accumulated enough positive outcomes to be more upbeat about its potential benefits.^24^ Addressing the Fourth International Medical Congress in Paris in late 1949, Viets presented the results of thirty-six patients, indicating that as experience increased, operative mortality rates fell to very low levels, but remaining guarded about the eventual value of the operation: ‘The key has been fitted to the lock and even partially turned, but the door is not open and what lies behind has not been even disclosed’.^25^

Other groups in the USA were also interested in the operation. The neurologist Kermit Osserman (1909–72) persuaded surgical colleagues to undertake the operation at Mount Sinai Hospital in New York.^26^ In 1942 O. T. ‘Jim’ Clagett (1908–90) undertook the first successful resection at the Mayo Clinic. Clagett and his colleague Lee Eaton (1905–58), a neurologist, presented a series of seventy-two of their patients and 142 non-operated controls to the American Neurological Association in 1949 and published the results the following year.^27^ They concluded that their controlled studies failed to demonstrate the value of thymectomy in myasthenia gravis.

## ‘Fortune favoured our efforts…’: Geoffrey Keynes’ thymectomies, 1942–52

In his memoirs, entitled *The Gates of Memory*, written in the late 1970s when he was in his early 90s, the English surgeon Geoffrey Keynes recounted how an interest in surgery of the thyroid had led him to become involved in attempts to treat myasthenia gravis surgically by removing the thymus gland. Keynes records that he was first asked to perform the operation in 1942 at the behest of Edward Arnold Carmichael (1896–1978), a physician at the National Hospital for Nervous Diseases in Queen Square, London. Carmichael, Keynes recalled, had seen a report of Blalock’s successful operation. Praising the ‘courage and will to live’ of his index patient, Keynes recounts his successful outcome: ‘Fortune favoured our efforts… After a normal convalescence she appeared to be cured and for a year worked ten hours a day as a land girl’.^28^

Nine months later Keynes’ early results were presented to the Section of Neurology of the Royal Society of Medicine by James Carson (1908–93).^29^ By this time twelve patients (most referred by Arnold Carmichael or Gordon Holmes at the National Hospital) had been operated on, of whom three had died. Carson noted that before the operation, all twelve had been ‘severely affected and… unable to work even with the help of prostigmin [neostigmine]’. Three patients had gone into remission, and no longer needed medication. Another three had improved, needing lower doses of medication. One patient had not been seen but was reported to have benefitted slightly, and two had not improved. The improvement had come early, being largely seen ‘by the time that the post-operative period is over’. ‘Our results’, Carson concluded, ‘and those published by Blalock and his colleagues indicate that extirpation of the thymus offers some prospect of recovery’. Commenting on Carson’s presentation of his patients, Keynes met potential criticism head-on: ‘The mortality of three out of twelve patients is heavy, though not perhaps heavier than was to be expected in the surgical treatment of so severe and obscure a disease as myasthenia gravis’, and concluded that ‘the results so far obtained by thymectomy for the severer forms of the disease are encouraging enough to warrant a further cautious trial, and we hope to present fuller and more extensive evidence of progress at some future date’. The response to this presentation was by no means unequivocally positive; the neurologist Russell Brain (1895–1966) and surgeon R. L. Galloway, in particular, noted that none of the three patients on whom the latter had operated had benefitted.

In his memoir Keynes admits that initial operative mortality was high – eight out of the first twenty-one patients died – but states that this was due not to deficiencies in the operation itself (or indeed in the operator) but to poor patient selection (many of the initial patients being presented for operation when the disease was too far advanced), or with a thymic tumour (which he believed carried a poor prognosis), or poor perioperative care by other doctors and nurses. When he had performed fifty-one operations, Keynes stated that ‘the time had come to describe and publicize the whole problem and its probable solution by surgery, so that the operation might be more widely practised and more sufferers relived from a truly terrible disease’. Keynes took thymectomy as his topic for the 1945 Hunterian Lecture at the Royal College of Surgeons^30^ and organised a meeting of the Royal Society of Medicine on the subject at the National Hospital in 1946. At the latter meeting, with thirty of his patients assembled in front of him, Keynes ‘challenged anyone to produce equal results by means of medical treatment or spontaneous remission in any comparable series of patients’.^31^

By 1949 Keynes had operated on 155 patients. Of the 120 patients who had survived, and whose outcomes he could report, thirty-nine were well, forty-three greatly improved, thirty-one were somewhat improved, and only ten were no better. In 1952, 100 of these patients were included in a review by R. T. Ross (1924–2017) – at that time a junior doctor at the National Hospital for Neurology and Neurosurgery – published in *The Lancet* in 1952.^32^ Keynes recalled this paper in his memoirs, stating that it contained the sober judgements of ‘an independent observer at the clinic in Queen Square, who had carefully and without bias examined’ his cases, contrasting this with the ‘serious breach of international good manners’ of an (unnamed) ‘American visitor’ who publicly doubted Keynes’ ability to remove the thymus in the middle of a tricky operation.

## Keynes vs. the Mayo Clinic

Keynes was lauded as a hero by the myasthenia community well after he stopped operating. The image of the (usually male) heroic surgeon, delving into the *terra incognita* of the thoracic cavity, and emerging having transformed the life of (often young and female) patient held a powerful aesthetic appeal for much of the twentieth century.^33^ Typically this story was played out in the context of lung disease (tuberculosis or cancer), or heart disease; however, the iconography was also applicable to myasthenia, where the transformative potential of surgery was illustrated by the change from the ‘snarl’ of the untreated (or untreatable) myasthenic into the broad smile of the treated patient. In his memoir, Keynes recollects widespread public interest in his successful treatment of the ‘Girl with the Frozen Smile’: one such report in *The Dundee Evening Telegraph* of 18 May 1948 reports how Keynes (‘brother of late Lord Keynes’) ‘restored the vanished smile to the face of Golda Murray, seventeen-year-old South African girl’ who was suffering from ‘a rare facial paralysis’. The *Telegraph* stated that though the operation had first been tried in America, ‘Mr Keynes developed and perfected the technique’, which was ‘so difficult that few surgeons practice it’.^34^

Every hero needs a villain. The villains of Keynes’ memoirs were the ‘medical team at the Mayo Clinic in Rochester, Minnesota, one of the most famous medical centres in the world’ who, as we have seen above, had publicly doubted the value of the operation. In *Gates of Memory* Keynes states that the ‘adverse opinion’ of the Mayo Clinic ‘soon spread all over the world and put me in a somewhat difficult position’. In 1954, Keynes was asked to deliver a lecture on thymectomy to the Medical Society of London.^35^ Doubtless, Keynes recalled with no little glee, they were expecting a retraction of his claims in the face of such opposition, but in fact, he unexpectedly found himself in the position of being able to tell his audience that the Mayo had ‘suddenly completely reversed its stand on the value of thymectomy, in a paper which now confirmed the results I had put forward nine years before, but which had been damned with fainter and fainter praise, even as the results seen in the actual patients got better’. Even worse, Keynes wrote, the perfidious Americans had announced this change of heart ‘almost *sotto voce*, in a highly specialised journal which would be read by no one except a few neurologists’.

According to Keynes’ account, it was not until 1976 that the Mayo eventually ‘came clean’, in a paper which unequivocally supported the value of the operation. By then, Keynes, concluded, ‘untold harm had been done to the victims of one of the most distressing and humiliating diseases to which human diseases are prone by the Mayo Clinic’s attempts to belittle the value of surgery’. Keynes, on the other hand, basked in the adulation of his patients, safe in the knowledge that he had been proved right, and that the debate about the value of thymectomy as a treatment for myasthenia was well and truly over.

Keynes parses this as a simple account of surgical innovation in which the self-evident truth that thymectomy did help treat myasthenia eventually prevailed, even in the face of the mighty Mayo Clinic. He portrayed himself as a salmon, doggedly swimming against the stream, knowing that despite the vicissitudes of the journey, his destination was correct and ultimate success certain. And yet, there are numerous examples in the history of medicine and science to caution against such an interpretation. Scientific or medical facts are contested: they are the outcome of controversies, not what determines or adjudicates them. Why, then, did the Mayo Clinic continue to use the technique, and then have ‘suddenly completely reversed its stand’? And why, if the matter was completely settled following the Mayo Clinic’s 1976 paper, as Keynes claims, did the *New England Journal of Medicine* need to publish the results of a ‘Randomized Trial of Thymectomy in Myasthenia Gravis’ in August 2016, fully forty years after the Mayo Clinic’s final admission of defeat, and over sixty years after the Clinic’s *volte-face*? The answers to these questions involve not only an understanding of the emerging technologies by which the success or otherwise of surgical interventions were starting to be judged in the 1940s and 1950s, but also of the social, financial, and professional circumstances in which the team at the Mayo Clinic, in particular, operated. In the crucial period between 1950–2, it is not too difficult to see why the team at the Mayo Clinic might have felt isolated. Keynes was reporting positive outcomes. Whilst the results of Viets’ team at Massachusetts General Hospital were not quite so good, Viets and Schwab were becoming much more positive about the potential of the procedure. The team at Johns Hopkins was more guarded, but Grob’s negative paper was yet to be published. Writing in 1955, Eaton and Claggett said that ‘we were disturbed by the differences between our results and conclusions, and those of other workers’.^36^ To understand why this discrepancy was disturbing, and what its implications were, we need to consider the social geography of surgery – and in particular thoracic surgery – in post-war America.

## Thoracic surgery in the post-war United States

Thoracic surgery was a relatively young speciality. Operating within the thoracic cavity was technically challenging, and the anaesthetic skill required the keep the patient alive whilst doing so was considerable because of the adverse consequences of opening the cavity on the lungs, which would collapse unless the operation was done in a negative pressure environment, or the lungs were kept inflated under positive pressure. In Europe, Ferdinand Sauerbruch (1875–1951) and his mentor Johann von Mikulicz (1850–1905) developed a negative pressure operating chamber; Sauerbruch brought the chamber to a meeting of the American Medical Association in 1908. Leaving the chamber in New York, it was used by the surgeon Willy Meyer (1858–1932), but it was clumsy and cramped; in 1928 it was broken up and sold for scrap. But it proved a stimulus for other approaches: at the Rockefeller Research Institute, Samuel Meltzer (1851–1920) developed intratracheal positive pressure ventilation, first used in 1910 at Mount Sinai Hospital, which proved to be a more successful solution to the problem.^37^

In his 1992 survey of the history and historiography of surgery, Chris Lawrence quotes Frederick Dennis (1850–1934), Cornell professor of clinical surgery, who wrote in 1905 that surgery demanded self-reliance, principle, independence, and determination, qualities which Dennis believed were conspicuous in the early settlers of the USA.^38^ Thoracic surgery, with its heroic delve into the hidden interior of the chest cavity, was a good fit for these qualities. As early as 1917 an American Association of Thoracic Surgeons (AATS) was created, even whilst several prominent nominees for its inaugural membership were serving in the armed forces in Europe.^39^ The AATS founded the *Journal of Thoracic Surgery* in 1931. The AATS had a strong East Coast of the United States, and more specifically New York bias, but included members from all over the United States, including the Mayo Clinic, at which the tradition of thoracic surgery dated back to 1915, when Dr William James Mayo (1861–1939) invited Samuel Robinson (1875–1947) to establish a thoracic surgery section at the clinic.^40^ Robinson’s tenure in Rochester was cut short because of illness, and after the war, Mayo asked Stuart Harrington (1889–1975) to continue his work. Harrington, who was president of the AATS in 1937–8, was succeeded in 1940 by Clagett, who expanded the subspecialty substantially in the post-war years and personally performed over 35,000 operations at the Mayo Clinic.^41^

Thoracic surgery developed rapidly as a sub-speciality in the United States between the 1940s and 1960s.^42^ Despite this, thymus surgery was never a major component of the work done by thoracic surgeons. Only three of the 900 pages of Richard Meade’s compendious 1961 *History of Thoracic Surgery* are devoted to the topic.^43^ Of Clagett’s extraordinary surgical count, no more than 500 (1.5%) can have been thymectomies. Prior to the Second World War the majority of the work of thoracic surgeons involved surgery for tuberculosis or other lung infections. Afterwards, cardiac surgery increasingly came to the fore, as extracorporeal circulation techniques were developed in the 1950s. The world’s first successful open heart operation was performed at the University of Minnesota Hospital in 1952.^44^ Cardiac surgery would come to define the speciality, but there was a short period in the 1940s and early 1950s in which thymectomy was the leading edge of thoracic surgery: novel, innovative, and noteworthy. In such a context, it is in the best interests of the team at the Mayo Clinic to be seen to be innovators, hence their early adoption of the technique, but an inconvenient and potentially embarrassing public variance of opinion over the value of a particular operation could not be allowed to deflect attention from the burgeoning successes of the speciality. This was an important impetus behind attempts to achieve closure in this debate. By 1952 thymectomy was no longer an exciting new operation, and heroic thoracic surgeons were already looking to move on. No one wanted to leave untidy loose ends behind them.

That there was genuine competition between leading American surgical centres for prestige, funding, and patients cannot be doubted. Competition and commerce were the beating heart of American culture. An unusually explicit view of how hospitals could advertise their services (and a clear demonstration that, however small-scale thymectomy was by comparison with other procedures, thoracic centres wanted to ensure their primacy in managing it) is seen on the dust jacket for Viets and Schwab’s 1960 book *Thymectomy for Myasthenia Gravis.* On the rear cover of the jacket there was a list of the publisher’s neurological offerings, preceded by a box containing the exhortation ‘Let These Neurologists Go to Work for YOU’. The front cover stated, ‘That the most comprehensive report to date on myasthenia gravis should emanate from the Massachusetts General Hospital was to be expected. The principal authors and their associates have observed and studied over five hundred examples of this uncommon disease since 1935.’ Noting that there had been ‘over one hundred and thirty-nine thymectomies’, the blurb explained that on ‘favorable [sic] patients, operated on under controlled conditions with proper anaesthesia and expert nursing, THE OPERATIVE MORTALITY HAS BEEN REDUCED ALMOST TO ZERO. These findings constitute a record of tremendous value…’. The message was clear: if your patient had myasthenia gravis, thymectomy was the solution, and Massachusetts General Hospital was the place to go.^45^ The Mayo Clinic, as a private foundation in a semi-rural setting far away from the affluent East Coast, relied upon its stellar reputation to attract patients from far and wide, and could not afford to be out of step with its competitors or pass up a potential source of patients.^46^

## Surgery and statistical analysis

In late 1952, the Mayo Clinic team put themselves through a potentially humiliating public *volte-face* on the subject of thymectomy. It is important to be clear that (contrary to what Keynes wrote in his memoirs) the data did not drive this process, even though in the 1952 retraction, and subsequent publications, Eaton allowed readers to infer that it was Keynes’ results that drove their reappraisal. We have seen how the competitive nature of post-war American surgery, and in particular the burgeoning speciality of thoracic surgery, may have driven the team at the Mayo to reconsider their position, but the way in which their retraction was presented, and the subsequent debates over the operation, turned not on the social and financial exigencies of the situation but on the statistical interpretation of surgical data.

When Keynes wrote that the Mayo team made their retraction in an obscure journal, he was being disingenuous, or at least unfair. The retraction was made at a national gathering of American neurologists: the annual meeting of the Association for Research in Nervous and Mental Diseases held in New York on 12 and 13 December 1952. It is true, however, that the proceedings of the meeting were not published until the following year, that the series in which it appeared was highly specialised, and might well have only been taken by a specialised neurological library such as that of the National Hospital, and that the title of the relevant volume, ‘Toxic and Metabolic Diseases of the Nervous System’, gave no hint that it might contain an article on the surgical treatment of myasthenia.^47^ It is not surprising that Keynes did not know about it for more than twelve months, but the Americans knew; the Association had over 700 members, there were more than fifty contributors to the proceedings of the 1952 meeting, and most of the leading protagonists in the thymectomy debate were there.

Eaton’s presentation was a delicate balancing act. He stated clearly that it had, until that point, been the view of his team that there was no evidence that thymectomy improved overall outcome in patients with myasthenia when comparing those who had had the operation with those who had not. He did not state explicitly what had driven the reappraisal, but he allowed his listeners (and readers) to assume that it was the results of other centres (Keynes in London, Viets in Boston, and so on) that had led them to do so. The discrepancy between the results of his group and others who operated was not due to poor surgical technique or peri-operative care but to patient selection. The Mayo team, Eaton noted, had included patients with thymic tumours in their original series. Once they were removed, the picture started to look much more favourable. Eaton took his audience through a process of removing those patients from the data, before going on to assess the impact of other factors (such as age, gender, and severity of illness) on outcomes after surgery, concluding that, after all, their outcomes were in line with those of other centres. ‘Much to the surprise of one of us’, said Eaton, referring to himself, ‘elimination of critically ill and older non-surgical patients from the previous comparison still indicated definitely superior results in surgically treated patients’.

Eaton mentioned, almost in passing, that their data had been subjected to statistical analysis, using methods ‘difficult for non-statisticians to comprehend’. No details were given of these analyses, perhaps because, as Eaton noted, their statistician had demurred from wholly supporting their conclusions, suspecting that their outcomes might yet be influenced by subtle discrepancies in the selection of patients for surgery, or by the positive psychological impact of having surgery. By mentioning this, Eaton ensured sufficient doubt to allow for a potential future reversion to their original point of view. This was important because, as he specifically stated at the outset, his view was that any improvement following thymectomy could not necessarily be attributed to the removal of the thymus; perhaps, he speculated, the operation itself stimulated ‘certain endocrine metabolic processes that account for the favourable result’. In the discussion that followed, Viets was magnanimous, praising Eaton for having the courage to announce his change of view in such a public forum. Eaton, in turn, thanked Viets for not having taken the opportunity to crow.

The capricious nature of myasthenia gravis clearly made it difficult to judge the success of thymectomy. Some patients with myasthenia would get better by themselves, and other patients would be able to control their disease with medication. In the 1940s, two statistical innovations had been employed to try to clarify the effect of surgical intervention: the stratification of outcomes, and the use of controls. Randomisation was not employed. Keynes had stratified his outcomes, but only for degrees of improvement – he did not allow for non-fatal worsening of the condition and did not have controls. The Mayo Clinic paper had utilised controls for their 1949 paper; doing so initially led them to question the value of the procedure. As Eaton’s presentation showed, however, using stratified outcomes and controls did not itself guarantee clarity, however; further processes of selection were necessary. Retrospective review of outcomes was potentially helpful in this process. Both Keynes and Viets had stopped operating on patients with thymic tumours because it had become clear that they did less well after the operation. Eaton achieved a relatively painless *volte-face* using the same technique. The issue of patient selection came to the fore between 1952 and 1960. It allowed the team at the Mayo – in a 1955 paper in the *American Journal of Medicine* (a publication that did pass over the desk of Keynes at the New End Hospital in Hampstead) – to criticise Keynes for his biases in patient selection, most notably his preference for operating on younger patients, more of whom were women.^48^ Both the Mayo Clinic and Massachusetts General teams had come to conclude that this patient group was more likely to benefit from the operation.^49^

In 1953, the team at Massachusetts General Hospital introduced a further statistical innovation – the case-control study – in which every patient who underwent thymectomy was prospectively matched with a patient of equivalent age, sex, severity, and duration of disease. These patients had all improved initially from the use of medication. Again, the figures seemed to indicate that the benefit of thymectomy was most apparent in women under the age of thirty-five, in whom remission occurred in 63% of those operated upon, vs. 34% of controls, and in whom mortality (including postoperative mortality) was 15% vs. 28% in the control group. The same was not true for male patients, in whom the control group fared better.^50^ Further analysis of patients five years later showed consistent results in women under forty.^51^ This conclusion was reiterated in a statistical analysis of 294 patients operated on in the UK from 1941 to 1958, and of the published American results, carried out by John Alexander ‘Iain’ Simpson (1922–2009), a clinical research fellow at the National Hospital, at the prompting of the neurologist Edward Arnold Carmichael. Simpson’s survey, first presented at the Royal Society of Medicine in 1956, and subsequently published in detail in *Brain* in 1958, suggested that any apparent discrepancies were due to the failure of the Americans to report thymoma cases separately, to the use of different criteria for classification and different methods of selection for operation, and specifically to the selection of unoperated cases as controls. He concluded that the results of all the series were essentially the same, allowing for the sampling errors of the smaller series. Simpson’s view was that the extent of improvement was more significant in women as they would otherwise have had a poorer prognosis than men.^52^

## Closure: ‘thymectomy is beneficial in myasthenia gravis’

Through the 1960s and 1970s consensus appeared to be approaching. The black box was closing: thymectomy was becoming a useful treatment for myasthenia. Developments in understanding the pathophysiology of myasthenia gravis tended to stabilise the place of thymectomy, at least initially. In 1960, Iain Simpson proposed the hypothesis that myasthenia was an autoimmune disease.^53^ Other publications around this time supported this possibility.^54^ The autoimmune hypothesis gained credence in the 1960s and 1970s, when immunization of rabbits with purified muscle-like acetylcholine receptors developed symptoms of myasthenia gravis, thus creating an experimental model for the disease (experimental autoimmune myasthenia gravis).^55^ Antibodies to the acetylcholine receptor were subsequently demonstrated to be present in a significant proportion of patients with the disease.^56^ The demonstrated involvement of the immune system in the aetiology of myasthenia provided an explanatory framework for the potential relevance of the thymus to the process, though the exact role of the organ remained obscure.

Cancer trials in the 1950s and 1960s, as Valier and Timmerman have shown, ‘often delivered at best marginal benefits, with endpoints more controversial and success more difficult to assess’; the ‘complex and contested data derived from… ever more complex and formal trials, along with rows over design and execution’ was nonetheless framed in such a way as to promote RCTs ‘as the axis of clinical research in the late 1950s’, a ‘technique… co-produced within the new political and organizational infrastructure of post-war British medicine, which itself was expected to benefit from the existence of the controlled trial’.^57^ As we have seen in the debates over the value of thymectomy, additional work was usually required to achieve consensus about what patients should be studied, and how best to define a control population. Protocol design ‘was an increasingly complex and contentious matter, as was the translation of trial findings into practice’.^58^ The problem of spontaneous remission, which bedevilled the myasthenia debate, also arose in trials of adrenocorticotrophic hormone (ACTH) and cortisone for leukaemia, in which, as Rigal shows, ‘methods and results were not easily comparable. In particular, the definition of remission still varied greatly from one centre to another’.^59^ Rigal notes how the French physician Jean Bernard (1907–2006) developed the use of “historical” controls, the analysis of hundreds of patients’ files, which provided him with an estimation of the frequency and duration of spontaneous remissions.

By the mid-1970s, there had still not been a randomised or prospectively controlled trial of thymectomy. However, ever more sophisticated techniques were being used to improve the choice of case controls. The Mayo Clinic, for example, was now using an International Business Machines (IBM) 370 computer to select medically treated controls who closed-matched operated patients. Their 1976 paper (the one mentioned by Keynes as the paper in which they finally ‘came clean’) concluded that long-term rates of survival and disease remission were higher in the operated group and that this held true for both male and female patients, regardless of age and duration of illness. The original sceptical stance of the Mayo was glossed over, the authors noting that ‘Keynes of Great Britain was an early and firm advocate of thymectomy, and Clagett and Eaton of this clinic supported the concept with some conflict in results related to the selection of patients, largely whether or not a thymoma was present’.^60^

Keynes’ successors in the UK continued to operate upon patients referred from the National Hospital and other London teaching hospitals. Other groups took up the operation and found it successful: Ronald Edwards (1910–83) and Andrew Wilson (1909–74) in Liverpool, for example, wrote in 1972 that they were ‘particularly convinced of the beneficial effects of the operation in women in the age groups of fifteen to forty years and of the successful return of these patients to normal household activities including those of rearing children’. Almost as an after-thought, they added, ‘Many of these patients have also resumed full-time or part-time work’.^61^

The uncontested benefit of thymectomy was moving along the trajectory from journals to monographs to textbooks. The Canadian neurologist Donald Calne (1936–), summarising the situation in his 1975 monograph *Therapeutics in Neurology*, stated that it was ‘now clear that thymectomy is beneficial in myasthenia gravis’,^62^ citing Simpson’s 1958 paper, as well as more recent reviews from Mount Sinai Hospital and Los Angeles.^63^ By the late 1980s, some authorities considered that ‘arguments as to whether thymectomy should be performed in myasthenia gravis have taken second place to the question of which is the appropriate surgical approach to the thymus’.^64^ Textbooks of neurology started to state as fact that thymectomy was beneficial for myasthenia. The 4th edition of the highly respected *Principles of Neurology*, edited by Raymond Adams (1911-2008) and Maurice Victor (1920-2001), for example, suggested that the operation was ‘recommended in practically all patients with uncomplicated myasthenia who are less than forty-five to fifty years of age and who, after a period of treatment with anticholinesterase drugs, are responding poorly’.^65^ In later editions the advice was even clearer: thymectomy was ‘advisable as an elective procedure in practically all patients with uncomplicated myasthenia gravis between puberty and approximately fifty-five years of age who, after a period of treatment with anticholinesterase drugs, are responding poorly’.^66^

## Failure to close: the afterlife of the thymectomy debate

By the time Keynes finished writing his memoirs, he could present the thymectomy debate as over and done with; a piece of medical history. However, one does not have to look very hard to find, if not actually dissenting, then at least cautiously sceptical voices through the 1960s and 1970s. Some of the voices were concerned about surgical mortality and effectiveness. In 1961, for example, the neurologist Fergus Ferguson (1899–1974), giving the presidential address to the Section of Neurology of the Royal Society of Medicine, noted that seven of the twelve of his patients that had been operated on in Manchester had died; yet Ferguson’s main concern about the procedure was not its dangers, but its unpredictability, and the difficulty of judging how helpful it really was in the context of the ‘natural tendency of myasthenia to remit and relapse’.^67^ The American neurologists Raymond Adams, Derek Denny-Brown (1901–81), and Carl Pearson (1919–81) wrote in 1962, ‘The consensus at present is that in myasthenia gravis without thymic tumour, remission following surgery is only slightly more frequent (30–40%) and slightly more complete than that which occur naturally’.^68^ Some teams, such as the one at Mount Sinai Hospital in New York, restricted the operation to certain patient populations, deeming it inadvisable in others.^69^ Commenting on the Mayo Clinic computer-matched study when it was presented at the Annual Meeting of the American Surgical Association in New Orleans in April 1976, Dr Earl Wayne Wilkins (1919–2020) of the Massachusetts General Hospital, while presenting data from their hospital and Mount Sinai broadly supportive of the Mayo Clinic’s conclusions, nonetheless asked the authors, ‘Is this good enough? Have we totally eliminated bias? Have we totally eliminated the possibility – as Henry Beecher, our chief of anaesthesia, used to challenge us – “is this a placebo operation?”’^70^

The ascendancy of the autoimmune hypothesis brought mixed blessings for thymectomy. Whereas it provided a theoretical basis for the relevance of removing an organ rich in lymphoid tissue, research demonstrated that improvement after thymectomy did not seem to correlate with a reduction in the level of antibodies in patients’ serum. In addition, on the basis of the hypothesis, the medical treatment of myasthenia expanded to involve the use of several agents to modulate or damp down the body’s immune response, including steroids, steroid-sparing drugs such as azathioprine, and other treatments such as plasma exchange and intravenous immunoglobulin infusions.^71^ As the standard medical treatment of myasthenia evolved in the 1970s and 1980s, the question of the position of thymectomy in the management of myasthenia was ripe for reappraisal.

This was the context in which the Milwaukee neurologists Michael McQuillen (1932–) and Mary Leone wrote a short piece casting doubt on the effectiveness of the operation for the 1977 Christmas edition of the prominent American journal *Neurology.* ^72^ McQuillen and Leone compared the remission rates following surgery in five studies with remission rates in those who had nonsurgical therapy, and in five studies (some of which were the same as the surgical studies). In total, they found a remission rate of 28% in 821 surgical patients vs. 24% in 985 nonsurgical patients. The paper was short, but its publication in the leading American general neurological journal ensured it was widely read. Three years later, in 1980, for example, the neurologist Lewis Rowland (1925–2017) called this paper ‘a brave but solitary voice, questioning the nature of the Emperor Thymectomy’s robes’. Rowland was sceptical about the authors’ doubts, noting that they confined themselves to remissions only, and might have over-emphasised the relevance of ‘possibly brief and early remissions in unoperated cases’. Nonetheless, Rowland concluded whether they were ‘absolutely correct or not’, they were not alone in being ‘disquieted by the lack of any prospectively controlled study of thymectomy’.^73^

This disquiet rumbled on in the background through the 1980s and 1990s. Reviews of controversies around the best surgical techniques and approaches for thymectomy started to throw doubt on the overall effectiveness of the intervention. The New York surgeon Alfred Jaretski III (1919–2014), writing in 1997, was particularly scathing about the quality of the available data: ‘The thymectomy literature is replete with confusing data, variously defined measurements, and unsupported conclusions. There must be a uniform classification of the severity of symptoms, a clearly defined ‘complete stable remission’ status (the most reliable measure of success), and the use of these measures in comparing thymectomy techniques. Most importantly, the analysis of data must adhere to accepted statistical principles’.^74^ Jaretski’s conclusions were very much in line with the emerging importance of evidence-based medicine (EBM), an approach to medicine that evangelised and proselytized the randomised double-blind controlled trial as the sole arbiter of clinical effectiveness. In 2000, the Quality Standard Subcommittee of the American Academy of Neurology published a practice parameter reviewing the evidence for thymectomy in myasthenia that concluded that any observed benefits could be ‘merely the results of the multiple differences in baseline characteristics between the surgical and non-surgical groups’.^75^ The neuromuscular group within the epitome of EBM – the Cochrane Collaboration – took on the topic in 2009, reporting four years later that no conclusions could be derived because no trials of sufficient quality had been reported.^76^

By the time the Cochrane Collaboration report was published, however, an RCT was underway. The impetus for this had come from the British neurologist John Newsom-Davis (1932–2007). Summarising the evidence for thymectomy in 2001, Newsom-Davis recast the likelihood of response in the context of the presence, or otherwise, of acetylcholine receptor antibodies and age. Seropositive, early onset patients (who were four times more likely to be women than men) were most likely to be helped; the evidence in seropositive, late-onset patients was, Newsom-Davis wrote, ‘not compelling’, and there did not appear to be ‘sufficient grounds for recommending thymectomy’ for seronegative patients.^77^ Following initial soundings at the meeting of the American Neurological Association in Boston in 2000, a series of further meetings, and feedback from grant submissions to the Medical Research Council in the UK, and the National Institute for Neurological Disorders and Stroke (NINDS) in the USA, funding was received from NINDS in October 2004. By 2008 over seventy centres in twenty-two countries were involved in the trial, which compared the effects of receiving steroids (prednisone) using an alternate-day dosing regime vs. steroids and thymectomy.^78^ Thirty-six centres eventually recruited 126 patients by 2012. Data were collected until 2015, and the results were published in the *New England Journal of Medicine* in 2016.^79^ Patients who underwent thymectomy had fewer symptoms of myasthenia over a three-year period than those who received prednisone alone; patients in the thymectomy group also had a lower average steroid requirement. Fewer patients in the thymectomy group than in the prednisone-only group required immunosuppression with azathioprine or were hospitalized for exacerbations. In the cautious terms of twenty-first-century EBM, thymectomy was vindicated.

## Conclusion

What conclusions, therefore, can we draw from the story of the controversies relating to the value of thymectomy for myasthenia gravis? Recent historiography of surgery has looked at the way that surgeons conceptualise their work and their own histories. From this viewpoint, the story of thymectomy for myasthenia is a seemingly simple tale of a surgical innovation created by Alfred Blalock, and refined by his successors. Geoffrey Keynes presents the story as one in which the self-evident correctness of a surgical intervention wins out against unbelievers and the technically inept. For historians, however, the thymectomy controversy provides an example within the history of surgery of what has long been accepted within the historiography of science, that is, that facts exist at the nexus of a series of scientific, technological, social, and cultural networks, none of which are fixed over time. As knowledge, technology, or social relationships change, so may the meaning of those facts in whose construction they have been employed.

In the same way that the inequities of power and class implicit in newspaper reports of Keynes’ ‘Girl with the Frozen Smile’ sit uncomfortably alongside twenty-first-century sensibilities, the idea of the prestigious autonomous surgeon, central to Keynes’ self-image (at least as portrayed in his memoirs) looks increasingly antiquated.^80^ Keynes, of course, was not entirely autonomous, but he was physically and institutionally separated from his collaborators. Although he records a close working relationship with Arnold Carmichael, sitting in on his outpatient clinics and even operating on occasions at the National Hospital, he was not on the staff at the National, and most of his operating was done at the New End Clinic in Hampstead.^81^ As Stephen Caspar, Simon Shorvon, and Alastair Compston have shown, the personal and institutional context of the development of neurology in Britain and, more specifically, at the National Hospital in London tended to separate rather than bring together physicians and surgeons.^82^ In addition, myasthenia was not a central research interest for Carmichael, unlike the American physicians involved in the controversy with Keynes. It may be no coincidence, therefore, that the institutional contexts that encouraged collaborative working between surgeons and physicians in post-war America were the ones in which one finds a subtle translation from a primary interest in the technical question of whether an operation was possible, and how best to do it, to a more nuanced question of whether an operation should be done at all.

Hence, while Keynes enthusiastically presented a traditional case series of outcomes, concentrating on operative complications and mortality, the early American publications on the operation were noticeably more cautious in tone. This may in part be explained by the fact that the American groups were all collaborations between thoracic surgeons and physicians with genuine research interests in myasthenia (Harvey and Grob at Johns Hopkins, Eaton at the Mayo Clinic, Ossermann in New York, and Viets at Massachusetts General), who saw at first hand the complexity of the condition, and the difficulties in judging who might best benefit from what remained a difficult and potentially dangerous operation. These teams were more innovative, employing stratification of outcomes, increasing attention to patient selection (including the development of case-control studies), and comparative studies, aiming to understand whether surgical or non-surgical interventions (such as irradiation of the thymus) were more effective. By the 1970s, further innovations such as computerization were starting to be employed. The introduction of these quantitative and statistical technologies illustrates how, as David Jones has noted, surgeons (who had employed quantitative techniques of analysis from as early as the start of the nineteenth century) increasingly came to utilize some, but not all, of the techniques that became embedded within the randomised control trial in the post-war era. Additionally, the increasing importance of teamwork can be understood as a consequence of the processes of the social construction of surgery, and in particular thoracic surgery, as a profession in post-war America, where as shown in this paper, innovation was perhaps particularly important as a driver for professional and commercial success in an environment that, overtly at least, was more competitive than the socialised national health system in which Keynes and his British colleagues worked.

New understandings, such as the discovery of the presence of anti-acetylcholine receptor (and other) antibodies in the blood of many patients with myasthenia, initially seemed to provide additional support for thymectomy by providing an obvious rationale for the excision of an immune-modulating organ as a potential therapeutic intervention. However, as Newsom-Davis’ views make clear, such discoveries ultimately worked to increase the amount of uncertainty about the operation, accelerating the collapse of consensus, the re-opening of the thymectomy ‘black box’, and the recruitment of the full weight of the technologies of evidence-based medicine to bear on the question, in the shape of the multicentre randomised control trial published in 2016, in which surgeons were at best part of a large, international team of investigators. In this sense, the debates around thymectomy provide a good case study of Tröhler’s contention that the therapeutic evaluation of surgery has required ever widening integration of information from other disciplines, and that one of the consequences of this process was the evolution of the prestigious autonomous surgeon into a multidisciplinary team.^83^ And yet, facts remain contingent and slippery. Even now, more than eighty years after Blalock’s first operation, uncertainty remains about the question of how best to deploy thymectomy in the management of patients with myasthenia gravis. As the title of the British neurologist Jon Sussman’s review of the 2016 trial put it, ‘Thymectomy: the more you know, the more you know you don’t know’.^84^

## Footnotes

1

Thomas Schlich , ‘Introduction: What is Special About the History of Surgery?’, in Thomas Schlich (ed), The Palgrave Handbook of the History of Surgery (London: Palgrave Macmillan, 2018), 1–24 .

2 The *Palgrave Handbook*, *op. cit.* (note 1) is an excellent summary of contemporary scholarship in this area.

3 D.S. Jones, ‘Surgery and Clinical Trials: the History and Controversies of Surgical Evidence’, in *Palgrave Handbook*, *op. cit.* (note 1), 479–501.

4

Andrew Warwick , ‘X-Rays as Evidence in German Orthopaedic Surgery, 1895-1900’, Isis, 96 (2005), 1–24 10.1086/49154316114797.

5

Sally Wilde and Geoffrey Hirst , ‘Learning from Mistakes: Early Twentieth-Century Surgical Practice’, Journal of the History of Medicine and Allied Sciences, 64 (2009), 38––7718669573 10.1093/jhmas/jrn055; Keynes’ account in his memoirs of surgeons visiting him to watch him remove the thymus shows that this process remained important well into the middle of the twentieth century and beyond, particularly for understanding and developing the technical aspects of the surgeons’ craft: Geoffrey L. Keynes , The Gates of Memory (Oxford: Clarendon Press, 1981). W. Bruce Fye’s history of the Mayo Clinic contains numerous examples of visiting surgeons appraising and learning from the Mayos and their successors: W. Bruce Fye , Caring for the Heart: Mayo Clinic and the Rise of Specialization (New York: Oxford University Press, 2015).

6

Ulrich Tröhler , ‘Surgery (Modern)’, in William F. Bynum and Roy Porter (eds), Companion Encyclopaedia of the History of Medicine (London: Routledge, 1993), 984––1028.

7

Ulrich Tröhler , ‘Statistics and the British Controversy About the Effects of Joseph Lister’s Systems of Antisepsis for Surgery, 1867-1890, Journal of the Royal Society of Medicine, 108 (2015), 280–726187307 10.1177/0141076815593720PMC4530413; Anna Greenwood , ‘Lawson Tait and Opposition to Germ Theory: Defining Science in Surgical Practice’, Journal of the History of Medicine and Allied Sciences, 53 (1998), 99–131 9585686 10.1093/jhmas/53.2.99; P. J. Kernahan , ‘Causation and Cleanliness: George Callender, Wounds, and Debates over Listerism’, Journal of the History of Medicine and Allied Sciences (2009) 64: 1–37 18583354 10.1093/jhmas/jrn043; Thomas Schlich , ‘No Time for Statistics: Joseph Lister’s Antisepsis and Types of Knowledge in Nineteenth Century British Surgery’, Bulletin of the History of Medicine, 94 (2020), 394–422 33416724 10.1353/bhm.2020.0070. See also the discussions of the use of statistics in Sally Frampton’s history of ovariotomy: Sally Frampton , Belly Rippers: Surgical Innovation and the Ovariotomy Controversy (London: Palgrave Macmillan, 2019).

8

Barron H. Lerner , The Breast Cancer Wars: Fear, Hope, and the Pursuit of a Cure in Twentieth-Century America, (Oxford: OUP, 2011)11949674; Sally Wilde , ‘See One, Do One, Modify One: Prostate Surgery in the 1930s’, Medical History, 48 (2004), 351–6616021930 10.1017/s0025727300007675PMC547921.

9 For discussions of the resistance of clinicians in general to the introduction of co-operative studies, and their unfamiliarity with the processes of randomization and data collection in particular, see Harry Marks , The Progress of Experiment: Science and Therapeutic Reform, 1900-1990 (Cambridge: Cambridge University Press, 1997).

10 The tensions and discordances created by this dichotomy are the subject of several studies, including David S. Jones , ‘Visions of a Cure: Visualization, Clinical Trials and Controversies in Cardiac Therapeutics, 1968-1998’, Isis, 91 (2000), 504–4111143786 10.1086/384853; Sally Frampton & Roger L. Kneebone , ‘John Wickham’s New Surgery: ‘Minimally Invasive Therapy’, Innovation and Approaches to Medical Practice in Twentieth Century Britain’, Social History of Medicine, 30 (2017), 544–6629713119 10.1093/shm/hkw074PMC5914418.

11

Helen Valier and Carsten Timmerman , ‘Clinical Trials and the Reorganisation of Medical Research in post-Second Word War Britain’, Medical History, 54 (2008), 493–510 10.1017/s0025727300002994PMC257045018958251.

12

Cynthia Tang and Thomas Schlich , ‘Surgical Innovation and the Multiple Meanings of Randomized Controlled Trials: The First RCT on Minimally Invasive Cholecystectomy’, Journal of the History of Medicine and Allied Sciences, 72 (2017), 117–4127667536 10.1093/jhmas/jrw027PMC5412068.

13 No detailed account of this controversy has hitherto been published though there is an outline of it in J.-C. Givel , ‘Historical Review’, in J.-C. Givel , M. Merlini , D. B. Clarke , M. Dusmet (eds). Surgery of the Thymus. Pathology, Associated Disorders, and Surgical Technique (Berlin: Springer-Verlag, 1990). A useful reference, particularly for the periods 1910–40 and 1970–90, is the review by the surgeon Joel D. Cooper , ‘History of Thymectomy for Myasthenia Gravis’, Thoracic Surgery Clinics, 29 (2019), 151–830927996 10.1016/j.thorsurg.2018.12.011.

14

William Gowers , A Manual of Diseases of Nervous System (London: J. & A. Churchill, 1886-1888).

15

Walter Russell Brain , Diseases of the Nervous System (London: Oxford University Press, 1933). In his widely praised and posthumously published 1940 textbook, Samuel Alexander Kinnear Wilson grouped myasthenia with other ‘conditions of unknown nature’ such as epilepsy, narcolepsy, and migraine: Samuel A. Kinnear Wilson , Neurology (London: Edward Arnold & Co, 2 vols, 1940).

16

Henry R. Viets , ‘The Miracle at St Alfrege’s’, Medical History, (1965), 9, 184–6. A useful review contemporary to the events presented in this paper is that of J. Hamilton Paterson in the April 1956 meeting of the Section of Neurology of the Royal Society of Medicine: ‘Discussion on Myasthenia’. *Journal of the Royal Society of Medicine*, 49 (1956), 789–98. See also J. C. Keesey , ‘Contemporary opinions about Mary Walker: a shy pioneer of therapeutic neurology’, Neurology (1998) 51: 1433–99818874 10.1212/wnl.51.5.1433; Stuart J. McCarter , et al., ‘The Mary Walker effect: Mary Broadfoot Walker’, The Journal of the Royal College of Physicians of Edinburgh, 49 (2019), 255–931497797 10.4997/JRCPE.2019.317; Mark W. Weatherall , ‘Walker’s miracle of science’, Lancet Neurology, 23 (2024), 34 .

17

Geoffrey L. Keynes , ‘The history of myasthenia gravis’, Medical History, 5 (1961), 313–2614455469 10.1017/s0025727300026612PMC1034650.

18

A. Blalock , et al., ‘Myasthenia gravis and tumors of the thymic region. Report of a case in which the tumor was removed’, Annals of Surgery, 110 (1939), 544–6117857470 10.1097/00000658-193910000-00005PMC1391425.

19

A. Blalock , et al., ‘The treatment of myasthenia gravis by removal of the thymus gland’, JAMA (1941) 117: 1529–33.

20

A. M. Harvey . ‘Some preliminary observations on the clinical course of myasthenia gravis before and after thymectomy’, Bulletin of the New York Academy of Medicine, 24 (1948), 505–2218875549 PMC1871303.

21

David Grob , ‘Course and management of myasthenia gravis’, JAMA (1953) 153: 529 10.1001/jama.1953.0294023000100113084409.

22

Henry R. Viets , ‘Myasthenia gravis’, JAMA (1945) 127: 1089–1096 .

23

G. Keynes , et al., ‘Symposium on the Surgical Treatment of Myasthenia Gravis’, Journal of the Royal Society of Medicine, 40 (1946), 600–410.1177/003591574603901003PMC218193221000447.

24

Henry R. Viets and Robert S. Schwab , Thymectomy for Myasthenia Gravis. (Oxford: Blackwell Scientific Publications, 1960).

25

Henry R. Viets , ‘Thymectomy in Myasthenia Gravis’, British Medical Journal, i (1950), 139–4710.1136/bmj.1.4646.139PMC203652915404170.

26

Kermit E. Osserman and Gabriel Genkins , ‘Studies in myasthenia gravis: review of a twenty-year experience in over 1200 patients’, The Mount Sinai journal of medicine, New York, 38 (1971), 497–537 4941403 .

27

L. M. Eaton and O. T. Clagett , ‘Thymectomy in the treatment of myasthenia gravis: results in seventy-two cases compared with one-hundred and forty-two control case’, JAMA, 142 (1950), 963–7.

28 Keynes, *op. cit.* (note 5).

29 ‘Discussion on Myasthenia Gravis and Thymectomy’, *Journal of the Royal Society of Medicine*, 37 (1943), 140–4. The Neurological Section of the Royal Society of Medicine was between 1920 and 1960 the main (often the only) forum for neurological debate in the UK: Stephen T Caspar , The neurologists. A history of a medical speciality in modern Britain, c. 1789-2000 (Manchester: Manchester University Press, 2014); Simon Shorvon and Alastair Compston , Queen Square: A History of the National Hospital and its Institute of Neurology (Cambridge: Cambridge University Press, 2019), 292–3.

30 Keynes, *op. cit.* (note 5).

31 At this meeting Keynes admitted for the first time that the presence of a thymic tumour adversely affected the outcome, making the operation “more difficult and dangerous”. The neurologist Purdon Martin, chairing the meeting, confessed to being disappointed that many of Keynes’ patients still needed to take medication: ‘Symposium’, *op. cit.* (note 23).

32

R.T. Ross , ‘Thymectomy in the Treatment of Myasthenia Gravis’, Lancet, 259 (1952), 802–3.

33 For the late 19^th^ century background to this image, see: Christopher J. Lawrence and Michael Brown , ‘Quintessentially Modern Heroes: Surgeons, Explorers, and Empire, c.1840-1914’, Journal of Social History, 50 (2016), 148–79; Michael Brown , ‘The ‘New World of Surgery’. Sepsis, Sentiment, and Scientific Modernity’, in Michael Brown , Emotions and Surgery in Britain, 1793-1912, (Cambridge: Cambridge University Press, 2022), 237–74. Much of the recent work of Agnes Arnold-Forster focusses on these and related issues; Agnes Arnold-Forster , Cold, Hard Steel. The myth of the modern surgeon (Manchester: Manchester University Press, 2023).

34 ‘Girl with the Frozen Smile’, *Dundee Evening Telegraph*, May 18, 1948.

35

Geoffrey L. Keynes , ‘Surgery of the Thymus Gland: second (and third) thoughts’, Lancet, i (1954) 1197–1202 10.1016/s0140-6736(54)92441-113164352.

36

L.M. Eaton , O.T. Clagett , J.A. Bastron , ‘The thymus and its relationship to diseases of the nervous system: study of 374 cases of myasthenia gravis and comparison of 87 eighty-seven patients undergoing thymectomy with 225 controls’, Research publications - Association for Research in Nervous and Mental Disease, 32 (1953), 107–-12413134638 .

37

J.B. Brodsky and H.J.M. Lemmens , ‘The History of Anaesthesia for Thoracic Surgery’, Minerva Anastesiologica, 73 (2007), 513–2417380101; A.P. Naef , ‘The mid-century revolution in thoracic and cardiovascular surgery: Part I’, Interactive Cardiovascular and Thoracic Surgery, 2 (2003), 219–2617670033 10.1016/S1569-9293(03)00130-0.

38

Christopher J. Lawrence , ‘Democratic, Divine and Heroic: The History and Historiography of Surgery’, in Christopher J Lawrence (ed), Medical Theory, Surgical Practice. Studies in the History of Surgery, (London: Routledge, 1992).

39

J.G. Scannell , ‘Historical Perspectives of the American Association for Thoracic Surgery. Willy Meyer (1858-1932)’, The Journal of Thoracic and Cardiovascular Surgery, 111 (1996), 1112 10.1016/s0022-5223(96)70242-88642838; W.G. Austen , ‘Eight Former Presidents of the AATS. The Boston connection’, The Journal of Thoracic and Cardiovascular Surgery, 98 (1989), 643–582682010 .

40

E.A. Gillaspie , F.C. Nichols , M.S. Allen , ‘Mayo Clinic: an Institutional History of General Thoracic Surgery’, Seminars in Thoracic and Cardiovascular Surgery, 27 (2015), 353–926811041 10.1053/j.semtcvs.2015.10.003.

41

A.P. Naef , ‘The mid-century revolution in thoracic and cardiovascular surgery: Part 2. Prelude to 20^th^ century cardio-thoracic surgery’, Interactive Cardiovascular and Thoracic Surgery, 2 (2003), 431–4917670091 10.1016/S1569-9293(03)00190-7. Fye, *op. cit.* (note 5).

42 The Board of Thoracic Surgery was founded in 1948; by 1960 it had certified approximately 1000 surgeons. New societies were formed, including the Southern Thoracic Surgical Association in 1954. By 1957, the attendance at the annual meeting of the AATS was over 1000, but the Society only allowed for 300 full members; discontent with this situation eventually led to the formation of a competing Society of Thoracic Surgeons in 1963, whose journal *Annals of Thoracic Surgery* first appeared in January 1965: R.G. Ellison , ‘Significant Events in the History of The Society of Thoracic Surgeons’, The Annals of Thoracic Surgery, 14 (1972), 577–604 4564851 10.1016/s0003-4975(10)65270-8.

43

Richard H. Meade , A History of Thoracic Surgery, (Springfield, IL: C. C. Thomas, 1961).

44 In 1952 Clagett’s protégé John Kirklin outlined to the Mayo Clinic’s Sciences Committee his plans to produce and experiment with a “mechanical heart in certain types of cardiac surgery”. Clagett himself never did open heart surgery, but he recognised that this was the future for his speciality. Two years earlier, in 1950, he had predicted that, if such a mechanical device could temporarily take over the functions of the heart and lungs, ‘there would be practically no limit to the surgical procedures that could be performed’. ‘I have the faith’, Clagett concluded, ‘to believe that the great days of surgery are ahead’.Fye, *op. cit.* (note 5), 206.

45 Viets and Schwab, *op. cit.* (note 22).

46 The Mayo Clinic also relied upon innovative graduate training programmes to attract high-quality doctors in training, including one of the earliest dedicated residencies in neurology: Christopher J. Boes , et al., ‘The Founding of the Mayo School of Graduate Medical Education’, Mayo Clinic Proceedings, 90 (2015), 252–6325659241 10.1016/j.mayocp.2014.12.008; Christopher J. Boes , et al., ‘Reciprocal Development and Progressive Responsibility: the History of the Mayo Clinic Neurology Residency’, Mayo Clinic Proceedings: Innovations, Quality & Outcomes, 4 (2020), 478–9810.1016/j.mayocpiqo.2020.06.006PMC755720833083697.

47 Eaton, Clagett, and Bastron, *op. cit.* (note 36).

48

L.M. Eaton and O.T. Clagett , ‘Present status of thymectomy in treatment of myasthenia gravis’, The American Journal of Medicine, 19 (1955), 703–1713268468 10.1016/s0002-9343(55)80012-5.

49

Robert Schwab and P. Passouant , ‘Les indications de la thymectomie au cours de la myasthénie’, La Presse Médicale, 60 (1952), 1501–313027081 .

50

Robert Schwab and Cy Leland , ‘Sex and age in myasthenia gravis as critical factors in incidence and remission’, JAMA, 153 (1953), 1270–313108586.

51 Viets and Schwab, *op. cit.* (note 22).

52 ‘Discussion’, *op. cit.* (note 16); J.A. Simpson , ‘An Evaluation of Thymectomy in Myasthenia Gravis’, Brain, 81 (1958), 112–4413523007 10.1093/brain/81.1.112.

53

J.A. Simpson , ‘Myasthenia gravis: a new hypothesis’, Scottish Medical Journal, 5 (1960), 419–36.

54

W.L. Nastuk , A.J. Strauss , and Kermit Osserman , ‘Search for a neuromuscular blocking agent in the blood of patients with myasthenia gravis’, The American Journal of Medicine, 26 (1959), 394–409 13626994 10.1016/0002-9343(59)90248-7; A.J. Strauss , ‘Autoimmunity in myasthenia gravis’, British Medical Journal, i (1963), 1245–610.1136/bmj.1.5444.1245-aPMC216662114275028; J.A. Simpson , ‘Myasthenia gravis as an autoimmune disease: clinical aspects’, Annals of the New York Academy of Sciences, 135 (1966), 506–165329432 10.1111/j.1749-6632.1966.tb45499.x.

55

J. Patrick and J. Lindstrom , ‘Autoimmune response to acetylcholine receptor’, Science, 180 (1973), 871–24706680 10.1126/science.180.4088.871.

56

A. Aharonov , et al., ‘Humoral antibodies to acetylcholine receptor in patients with myasthenia gravis’, Lancet, 2 (1975), 340–251144 10.1016/s0140-6736(75)92779-8.

57 Valier and Timmerman, *op. cit.* (note 11), 502. This process can be understood as an example of a wider transformation in British medicine from late Victorian individual competition to the corporate, managerial system embodied within the new NHS: Steve Sturdy and Roger Cooter , ‘Science, Scientific Management, and the Transformation of Medicine in Britain c.1870–1950’, History of Science, 36 (1998), 421–6611620554 10.1177/007327539803600402.

58 Valier and Timmerman, *op. cit.* (note 11), 501. See also: Carsten Timmermann , A History of Lung Cancer. The Recalcitrant Disease (London: Palgrave Macmillan, 2013). By the early 1960s, features such as clearly stated inclusion and exclusion criteria, pre-study tests, a clear description of the treatment plan, randomization by the sealed envelope technique, and statistical analysis by sequential methods were all included in the VAMP trial in leukaemia: Peter Keating and Alberto Cambrosio , Cancer on Trial. Oncology as a New Style of Practice (Chicago: University of Chicago Press, 2012).

59

Christelle S. Rigal , ‘Neo-clinicians, Clinical Trials, and the Reorganisation of Medical Research in Paris Hospital after the Second World War: the Trajectory of Jean Bernard’, Medical History, 54 (2008), 511–-53410.1017/s0025727300003008PMC257044418958252.

60

J. M. Buckingham , et al., ‘The Value of Thymectomy in Myasthenia Gravis: A Computer-assisted Matched Study’, Annals of Surgery, 23 (1976), 453–810.1097/00000658-197610000-00008PMC13454411015890.

61

F. Ronald Edwards and Andrew Wilson , ‘Thymectomy for Myasthenia Gravis’, Thorax, 27 (1972), 513–65083719 10.1136/thx.27.5.513PMC470538.

62

Donald Calne , Therapeutics in Neurology, (Oxford: Blackwell Scientific, 1975).

63

A.E. Papatestas , et al., ‘Studies in Myasthenia Gravis: Effects of Thymectomy. Results on 185 Patients with Thymomatous and Nonthymomatous Myasthenia Gravis, 1941-1969’, The American Journal of Medicine, 60 (1971) 465–7410.1016/0002-9343(71)90336-65572596; D.G. Mulder , et al., ‘Surgical management in myasthenia gravis’, The Journal of Thoracic and Cardiovascular Surgery, 63 (1972), 109–135008108 .

64

A.E. Papatestas , et al., ‘Effects of Thymectomy in Myasthenia Gravis’, Annals of Surgery, 206 (1987), 79––883606235 10.1097/00000658-198707000-00013PMC1492935. For a brief review of these discussions from the point of view of three cardiothoracic surgeons, see J.R. Sonett , M.J. Magee and L. Gorenstein , ‘Thymectomy and myasthenia gravis: A history of surgical passion and scientific excellence’, The Journal of Thoracic and Cardiovascular Surgery, 154 (2017), 306–928479053 10.1016/j.jtcvs.2016.12.074.

65

Raymond D. Adams and Maurice Victor , Principles of Neurology, 4th *edition*, (New York: McGraw-Hill Professional, 1989), 1158 .

66

Allan H. Ropper and Maurice Victor , Adams and Victor’s Manual of Neurology, 7th *edition*, (New York: The McGraw-Hill Companies, 2001), 1545 .

67

Fergus R. Ferguson , ‘A Critical Review of the Clinical Features of Myasthenia Gravis’, Journal of the Royal Society of Medicine, 55 (1962), 49––5210.1177/003591576205500114PMC189639713892331.

68

Raymond D. Adams , Derek Denny-Brown , and Carl M. Pearson , Diseases of Muscle: a Study in Pathology, (London: Henry Kimpton, 1962).

69

I. Kreel , et al., ‘Role of Thymectomy in Myasthenia Gravis’, Annals of Surgery, 165 (1967), 111–176018288 10.1097/00000658-196701000-00015PMC1617365.

70 Buckingham *et al.*, *op. cit.* (note 60).

71

J. C. Keesey , ‘A History of Treatments for Myasthenia Gravis’, Seminars in Neurology, 24 (2004), 5–16 15229787 10.1055/s-2004-829584.

72

Michael P. McQuillen and Mary G. Leone , ‘A treatment carol: thymectomy revisited’, Neurology, 27 (1977), 1103–6563009 10.1212/wnl.27.12.1103.

73

Lewis P. Rowland , ‘Controversies about the treatment of myasthenia gravis’, Journal of Neurology, Neurosurgery, and Psychiatry, 43 (1980), 644–597400825 10.1136/jnnp.43.7.644PMC490631.

74
A. Jaretski, ‘Thymectomy for myasthenia gravis: Analysis of controversies regarding technique and results’, Neurology, 48 (1997, suppl 5): 52S–63S .

75

G.S. Gronseth and R. J. Barohn , ‘Practice parameter: thymectomy for autoimmune myasthenia gravis (an evidence-based review): report of the Quality Standards Subcommittee of the American Academy of Neurology’, Neurology, 55 (2000), 7––1510891896 10.1212/wnl.55.1.7.

76

G. Cea , M. Benatar , R. J. Verdugo , R. A. Salinas , ‘Thymectomy for non-thymomatous myasthenia gravis’, Cochrane Database of Systematic Reviews, 10 (2013), CD00811110.1002/14651858.CD008111.pub2PMC1208387724122674.

77

John Newsom-Davis , ‘Disorders of the neuromuscular junction’, in Neil Scolding (ed), Contemporary Treatments in Neurology (Oxford: Butterworth-Heinemann, 2001), 158–69.

78

J. Newsom-Davis , et al., ‘Status of the thymectomy trial for nonthymomatous myasthenia gravis patients receiving prednisone’, Annals of the New York Academy of Sciences, 1132 (2008), 344–718567886 10.1196/annals.1405.014.

79

G.I. Wolfe , et al., ‘Randomized Trial of Thymectomy in Myasthenia Gravis’, The New England Journal of Medicine, 375 (2016), 511–2227509100 10.1056/NEJMoa1602489PMC5189669.

80 Arnold-Forster, *op. cit.* (note 33).

81

Geoffrey Keynes , ‘The history of myasthenia gravis’, Medical History, 5 (1961), 313–2614455469 10.1017/s0025727300026612PMC1034650

82 Stephen T Caspar, *op. cit.* (note 29), 105–6, 144–5. There was no neurosurgery department at the National Hospital at all between 1939 and 1946. Collaborative working between physicians and surgeons was limited in the post-war period because of disputes between senior staff members at the hospital: Shorvon & Compston, *op. cit.* (note 29), 317–20.

83 This paper has concentrated on the importance of collaboration between physicians and surgeons, but there are many other important players to whom equal attention could have been given, including anaesthetists, nursing staff, laboratory scientists, administrators, and so on. There is also interesting and important work to be done around the cultural impact of high profile cases of ‘rogue’ doctors and surgeons such as the GP Harold Shipman or the surgeon Ian Paterson on driving team working in medicine and surgery as a solution to perceived problems relating to patient safety.

84

Jon Sussman , ‘Thymectomy: the more you know, the more you know you don’t know’, Practical Neurology, 16 (2016), 426–728120786 10.1136/practneurol-2016-001543.

